# Social prescribing needs and priorities of older adults in Canada: a qualitative analysis

**DOI:** 10.24095/hpcdp.44.9.03

**Published:** 2024-09

**Authors:** Cindy Yu, Simran Lail, Sandra Allison, Srija Biswas, Paul Hebert, Sonia Hsiung, Kate Mulligan, Michelle L. Nelson, Marianne Saragosa, Vivian Welch, Kiffer G. Card

**Affiliations:** 1 The GenWell Project, Toronto, Ontario, Canada; 2 Simon Fraser University, Vancouver, British Columbia, Canada; 3 University of British Columbia, Vancouver, British Columbia, Canada; 4 Canadian Institute for Social Prescribing, Canadian Red Cross, Toronto, Ontario, Canada; 5 Centre for Research, University of Montreal Hospital Research Centre, Montral, Quebec, Canada; 6 Dalla Lana School of Public Health, University of Toronto, Toronto, Ontario, Canada; 7 Science of Care Institute, Lunenfeld-Tanenbaum Research Institute, Science Health System, Toronto, Ontario, Canada; 8 Institute of Health Policy, Management and Evaluation, University of Toronto, Toronto, Ontario, Canada; 9 Bruyre Research Institute, Ottawa, Ontario, Canada; 10 School of Epidemiology and Public Health, University of Ottawa, Ottawa, Ontario, Canada

**Keywords:** social prescribing, qualitative research, older adults, social determinants of health, social needs

## Abstract

**Introduction:**

Social prescribing (SP) is a holistic and collaborative approach to help individuals access community-based supports and services for their nonmedical social needs. The aim of this study was to assess the needs and priorities of Canadian older adults (aged 55 years and older), with a focus on optimizing SP programs for those who are systemically disadvantaged and socially marginalized.

**Methods:**

Semistructured focus groups (N=10 groups, 43 participants) were conducted online via Zoom with participants from across Canada. Data transcription and thematic analysis were completed in NVivo. Analyses were informed by self-determination theory.

**Results:**

Our results suggest that older adults desire SP programs that respect their ability to maintain their autonomy and independence, aid and facilitate the development of connectedness and belonging, are built on a foundation of trust and relationship-building in interactions with providers and link workers, and prioritize the person and thus personalize SP to the unique needs of each individual.

**Conclusion:**

SP programs should be informed by the values of older adults. As work is currently underway to formalize and scale SP in Canada, personalizing these programs to the unique circumstances, needs and priorities of participants should be a top priority.

**Highlights**

- Structurally disadvantaged and socially marginalized older adults want social prescribing (SP) programs that respect their autonomy and independence, boost their social connections with others and help them regain a sense of belonging in their community.
- Trust and a solid relationship with a link worker or health care provider are of utmost importance.
- Each older adult is unique, necessitating personalized supports and resources, particularly if they are structurally marginalized and socially disadvantaged.
- SP implementation in Canada should aim to meet older adults’ needs for autonomy, relatedness and competency in order to be effective.

## Introduction

***Social prescribing as a holistic intervention***

Social prescribing (SP) is a holistic approach to improving health and well-being by addressing participants’ nonmedical, health-related social needs, such as poor social integration, housing and food insecurity and poor mental health.^1^,^2^ It accomplishes this goal by providing a formal framework for health care providers and interprofessional community providers to refer clients to local, community-based, nonmedical services and supports that can address the client’s personal well-being, interests and needs through a person-centred and collaborative approach. In doing so, SP creates strong integration between medical care and community care systems, while also empowering clients to engage with their community and actively participate in improving their own health.^3^ Successful implementation of a SP program involves follow-ups to reduce barriers to access and ensure that supports were appropriate and beneficial to the client. Thorough program evaluation of SP interventions is also key to ensure they are meeting participants’ needs.^4^-^6^

***History and local adaptation of social prescribing programs***

The idea and implementation of SP originated in England in the mid-1980s to early 1990s to direct patients to local nonclinical services. Since then, SP has gained traction in many countries.^7^ In Canada, SP has also been gaining momentum, spurred by a growing body of evidence highlighting the importance of addressing social determinants of health for overall health and well-being.^8^,^9^ The way that SP is implemented is context-dependent and may differ across jurisdictions. Such differences include the scale and scope of services offered and how health and social services are integrated.^7^ Therefore, it is important to identify the needs of local program participants when scaling SP programs. 

***Social prescribing needs of older adults***

Although social isolation and loneliness are detrimental to the well-being and health of all populations, they are an underappreciated risk for older adults in particular.^10^,^11^ Older adults are more likely to experience risk factors that bidirectionally worsen social isolation and loneliness, such as chronic illness, loss of family members and living alone.^12^ Evidence suggests social isolation is linked to major health risks as well, including premature mortality.^13^ Older adults in marginalized, underserved populations are particularly impacted by the adverse effects of social isolation and loneliness on overall well-being and quality of life.^12^,^14^

In order to ensure that SP pathways, as well as the supports and services they link clients to, can provide benefit to those in greater need of the programs, it is crucial to understand the priorities of older adults who face structural disadvantages and social marginalization; in other words, older adults who may be limited in their privileges in the current system and social structure due to their race, gender, sexuality, age, disability, isolation and socioeconomic status.^15^ Given the person-centred nature of SP, client participation in co-designing and co-production (i.e. development of service activities through mutual understanding and agreement of service users [older adults] and service providers [health care and community providers]) is an integral element of the sustainability of SP.^16^

Research on SP has demonstrated that it has the potential to aid older adults in meeting their health and well-being needs.^11^,^17^ Bhatti et al. explored how SP facilitates positive outcomes in patients (aged 26–81 years) from 11 community health centres across Ontario, and found patients reported that engaging in SP satisfied their psychological needs for autonomy, competence and relatedness.^18^ In their literature review, Rothe and Heiss identified the need for link workers that take an active and supportive role for individuals with psychosocial needs, and for some with physical or mental illness.^19^

Furthermore, most studies indicated the importance of referring participants to activities that meet personal preferences and identity needs. Wildman et al. interviewed participants (aged 40–74 years) living in a socioeconomically deprived area in North East England.^20^ Although participants reported improvements in social connection and condition management, they also experienced difficulties related to multimorbidity, family circumstances and social, economic and cultural factors, outlining the importance of more complex SP interventions for those facing a multitude of social disadvantages and structural marginalization. The role of a strong and supportive relationship with an accessible link worker was particularly important for this study sample as well.

***Study aims and objectives***

Currently, there is limited research on the SP needs, attitudes and beliefs of older adults from diverse backgrounds.^21^ This understanding plays an important role in introducing and advancing SP in Canada. Our study aimed to explore the unique needs and priorities of older adults who experience systemic disadvantage and social marginalization. The study specifically focussed on gathering insights into the following: (1) older adults’ experiences with and interest in SP; (2) their comfort with their primary health care provider; (3) their comfort with other community providers; (4) qualities they would like in a link worker; and (5) barriers that may impede their participation. 

## Methods

***Ethics approval***

Study procedures were approved by the Research Ethics Board of Simon Fraser University (REB #30001382).

***Theoretical framework***

This study is informed by self-determination theory (SDT), which advances the idea that people who are enabled to self-determine their actions are more likely to experience greater well-being and motivation for change. The theory posits that self-determination requires the satisfaction of three psychological needs: (1) autonomy (i.e. a sense of control over one’s behaviours, having choice and decision-making power in what is important); (2) competency (i.e. the ability to achieve what one sets out to do effectively and have influence on outcomes); and (3) relatedness (i.e. social connectedness and belonging, and feeling understood, cared for and valued by others).^22^ SDT has previously been implemented in research into healthy aging among older adults^23^ as well as in understanding and improving SP specifically.^18^,^24^,^25^

***Participant recruitment and data collection***

This qualitative study used semistructured focus groups and thematic analysis to explore older adults’ attitudes toward and experiences with SP. A focus group is a specific form of interview that encourages engagement among participants, with the interviewer serving as a facilitator of discussion.^26^ Focus groups were conducted via Zoom, lasted anywhere between 60 and 90 minutes, and included between 3 and 7 participants per group, alongside a facilitator. Facilitators (CY, SL) underwent training with lab colleagues in practice focus groups, prioritizing practising open facilitation skills and awareness of sensitive topics. 

Focus group questions were designed with input from all authors, and are presented in Table 1. All focus groups were conducted in English. 

**Table 1:** Focus group questions on social prescribing needs and priorities of older adults in Canada

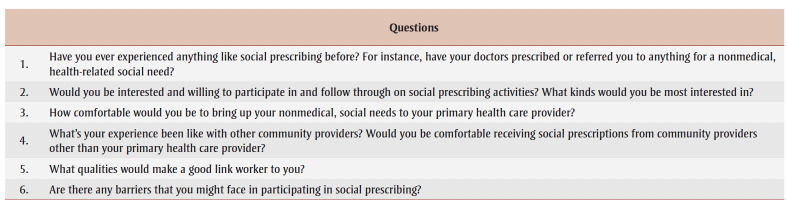

All participants gave informed consent. Older adults were recruited through a previously conducted online survey focussing on health care needs and utilization and SP attitudes among older adults (aged 55years and older who resided in Canada). The online survey was promoted via paid advertisements on Facebook, Instagram, Twitter and Google Ads, and through frontline health care services, and all participants were enrolled in a prize draw for CAD 200 in cash. The online survey introduced participants to the idea of SP and outlined important differences in health and overall well-being outcomes by demographics and lived experience.

Eligibility was limited to older adults who experienced at least one of the following forms of social marginalization and structural disadvantage, with priority given to those who reported multiple forms: fair or poor health; disability; racialized group status, newcomer status or Indigenous identity; 2SLGBTQI+ identity; low household income (< CAD 30000 per year); and social isolation or being homebound. Of note, these factors may disadvantage and marginalize individuals to differing degrees, and every participant in this study experienced at least one form.

After the email invitation, participants met via Zoom and participated in semistructured focus groups exploring experiences with and attitudes toward SP. Prior to the discussion, information about SP was presented to ensure participants understood what the intervention was and what it typically entailed. Data collection occurred via Zoom’s video recording and audio transcription for cloud recordings features. Minor inaccuracies in the transcription were corrected by manually reviewing video recordings of the focus groups. 

***Data analysis***

Data analysis was performed in NVivo Version 11,^27^ used a thematic approach developed by Braun and Clarke^28^ and applied SDT.^22^ The thematic analysis process began with multiple readings of the transcripts to gain a comprehensive understanding of the data. Initial codes were created based on the patterns, insights and themes that emerged from the data. Codes were then organized into broader themes over multiple rounds of mapping and connecting initial codes. These broader themes were reviewed and refined through iterative processes of analysis until final themes were established. Data analysis was considered complete when theme and data saturation were achieved. Quotations from transcripts that highlighted salient points were selected for inclusion in the analysis. 

## Results

The demographic information of those who participated in the focus groups is provided in Table 2.

**Table 2:** Older adult focus group participant demographics

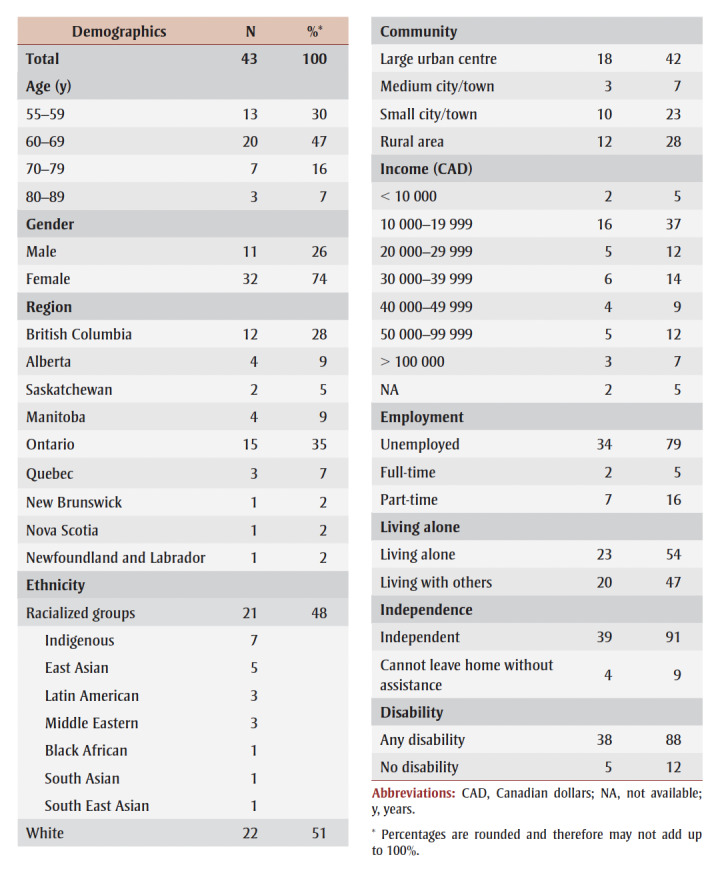

Thematic analysis of the results of these semistructured focus groups identified several key themes: (1) the importance of considering older adults’ sense of autonomy and independence; (2) a sense of connectedness and belonging, trust and relationship-building with providers; and (3) the uniqueness of each individual’s personal identity and how it shapes their needs, wants and barriers with respect to SP. These themes underlie our understanding of the five categories of discussion, namely:

- (1) older adults’ experiences with and interest in SP;
- (2) their comfort with their primary health care provider;
- (3) their comfort with other community providers;
- (4) the qualities they would like in a link worker; and
- (5) the barriers that may impede their participation.

Each of these categories is examined in greater depth below, and includes quotations from the focus group participants.

***1. Older adults’ experiences with and interest in SP***

Older adults’ experiences with and interest in SP varied widely. Very few knew about SP or had experience with it, whether formally or through practices with SP components. The ones who had experience or knowledge of it had often heard about nonmedical supports through various providers, such as doctors and community health workers, among others, for health-related challenges (e.g. post-surgery) but also life changes and transitions (e.g. loss of a spouse, divorce) and overall mental health and stress. SP-like interventions fell in the categories of social groups, health and wellness, community resources, and career and financial support. Notably, most SP experiences were not called SP, but were person-centred, nonmedical referrals, more broadly speaking. These results suggest there is a need to raise awareness about SP, and to formalize the concept of SP. 

Willingness to participate in SP also varied widely among participants. Those uninterested in SP often mentioned their contentment and satisfaction with their current interpersonal connections and communities (e.g. family ties, close friends and acquaintances) and preference for solitude. Importantly, underlying both reasons were two core factors: that their present state was both healthy and independent. If either of these two factors deteriorated, older adults often indicated they would consider SP then. Notably, healthiness and independence were not strongly age-dependent, as some individuals in their eighties perceived themselves to be physically and cognitively fit and capable:

I’m feeling very thankful for my own good health because I really don’t have major health problems, and I’m able to be very active physically and in the community. (Woman, aged 81, small city/town resident) 

Of those interested in SP, categories of SP activities of interest included holistic health, wellness and fitness, arts and culture, education and personal development, social groups and community, and nature and outdoor activities. The diversity of SP activities of interest, some of which were even opposed (e.g. something online vs. something outside of home), suggest the need for a person-centred and individualized approach to SP. As one participant noted, “Each person is unique and their needs are unique” (woman, aged 64, rural area resident).

For those who wanted to participate in SP, a prominent theme that arose was the desire to keep active and engaged in mind and body and socially. These individuals expressed a strong need for social connection and companionship, often due a lack in current social networks or family ties. Some preferred connections with others with similar backgrounds or interests, while others were interested in diverse interactions, including intergenerational and animal companionship. The desire to “live life again” underscored their reasons for SP participation: 

Yes, I would be interested in participating and looking more for things where there is some human connection. We can live quite solitary lives, and it would be helpful to integrate back into a community. (Woman, aged 72, small city/town resident) 

***2. Comfort with primary health care providers***

Primary health care providers and link workers play integral roles in the SP process, and thus the relationship between older adults and their providers and link workers is an essential factor to consider. Older adults were asked questions about their comfort level with their primary health care provider, as well as to identify the qualities most valued in a potential link worker. Individuals’ comfort levels discussing nonmedical, health-related social needs with their health care providers varied significantly. In the focus groups, participants often discussed their overall relationship with their provider rather than specifically addressing whether they were comfortable discussing social needs with their health care provider. This highlights the importance of the overall client–provider relationship in shaping social prescribing processes. 

Older adults with positive relationships cited genuine care, trust and good rapport as essential factors. Their health care providers did not stick only to the medical model but were willing to work outside of it, asking about emotions as well as life; the older adults felt like their health care provider genuinely cared and had good intentions, evidenced by not being in a rush, listening, following up and being personable. These relationships were long-lasting and built on a foundation of trust: 

I do trust my doctors. I’m lucky to have a really good family doctor and a really good pain doctor. My pain doctor especially isn’t afraid to go outside of medical to, you know, like he recommended that mindfulness thing, and he’s always searching for new ideas, that aren’t necessarily involving taking drugs. (Woman, aged 69, small city/town resident) 

Older adults with negative relationships with their health care providers indicated that the lack of those same factors (i.e. genuine care, trust and rapport) had a detrimental effect on their comfort level with their health care providers. Their health care providers often addressed solely medical concerns (e.g. renewing prescriptions), were dismissive of emotional concerns and didn’t ask questions to probe for deeper, underlying issues. In these cases, there was no trust or connection between the older adult and their health care provider, potentially because the provider was too busy (e.g. one concern per visit, phone first); the provider did not seem to genuinely care (e.g. often gave up, seemed impersonal and intimidating, did not follow-up, lacked empathy); or the provider was forceful as an authority (i.e. someone “who knows best”):

But there are signs that specifically say, please be advised that doctors can only help you for one question per visit per day. It’s intimidating. And then we don’t feel comfortable taking up space that you’re not allowed. (Woman, aged 58, small city/town resident) 

Even among individuals with positive relationships, some still felt uncomfortable discussing social needs because it was an unfamiliar topic to health care. This suggests the need to normalize such conversations. As one woman noted, “I have a regular doctor that I see at the clinic that I consider my doctor; but he’s also very much under a time crunch and time pressure. And so he doesn’t ask, and I don’t tell” (woman, aged 55, large urban centre resident).

For other older adults, external factors also affected their relationships with their health care provider, such as not having a primary health care provider at all, having a provider who was entirely remote and thus would not be aware of community resources and support, and having only newly established a relationship with the provider. For older adults with new relationships, a common trend was that they had a long-standing relationship with their previous provider, who had retired, and now needed more time to build trust and connection with their new one.

Rural individuals either reported positive relationships due to their provider being personable and involved in their community, or negative ones, due to their provider being in their town on a short-term contract (e.g. 2 years): “We rotate through doctors so often in [rural areas] that I have a feeling that 2 years from now, I’m going to have a new doctor again. So I’d have to build a totally new relationship” (woman, aged 61, small city/town resident). 

Some individuals felt that SP did not belong in the realm of the medical system at all, as health care providers and the medical system were already overburdened, but also because it wasn’t their expertise. 

These findings suggest the essential nature of a positive and strong relationship between older adults and providers before questions of SP can even be addressed. Without a positive relationship, introducing new and unfamiliar topics such as loneliness, rather than purely medical topics, will likely be unproductive. 

***3. Comfort with community providers***

Older adults were generally more comfortable discussing nonmedical, health-related social needs with community providers instead of their primary health care providers. Comfort level depended on personal experience, and varied across a range of community providers (e.g. community mental health workers, social workers). Major reasons for comfort stemmed from personal experience, trust and familiarity, accessibility and availability, and perceived integration within the community. 

Some concerns were raised regarding the professionalism and expertise of these community providers, given the diversity in professional training and backgrounds of potential SP providers. Some participants preferred that all community providers receive SP-specific formal training or accreditation and that community providers had a clear understanding of their practice boundaries, scope and limitations: “I would have no problem with the community providers so long as they were attuned at some educational level or experiential level to what my specific needs may be” (woman, aged 58, large urban centre resident).

Furthermore, some participants from rural areas lacked experience with community providers because in their rural communities there was a lack of community providers to be referred by, and resources to be referred to. 

***4. Qualities in link workers***

Compared to topics of SP interest and provider relationships, there were clearer core qualities that participants wanted in a link worker. Every discussion touched on core qualities of care, empathy and good communication skills. They wanted to interact with a link worker who was nonjudgmental of the participant’s concerns, was open-minded and who could genuinely care about the individual. Empathy could be demonstrated by a link worker being personable and demonstrating genuine curiosity, by being a good listener, by asking insightful questions and by being engaged in the conversation: 

I want to be heard. I want to be listened to. I want somebody who can check for understanding along the way, because I have that much more respect for the person who says, “Okay, this is what I’ve heard from you.” I want a meaningful conversation that goes both ways to know that that person held space for me. That’s meaningful. That puts me in a relaxed position where I go, “Oh, thankfully, I finally got somebody who’s listening to me!” (Woman, aged 59, large urban centre resident) 

Following empathy, creativity and knowledge about SP and referral resources and supports were also key criteria. Participants wanted a link worker who could think outside of the box, think beyond a set rubric or guideline and consider the unique needs of the person in front of them. They wanted a link worker who was connected to the community, who demonstrated competency and resourcefulness and who could understand the diverse needs older adults might bring to them. Ideally, the link worker would also be an expert in the field and have professional training and qualifications to meet this role seriously: 

They need to have a broad understanding of the community they live in or work in … which is sometimes not the community they live in. Be trained in delivery of that information in a nonjudgmental and broad-minded and sensitive way. Additionally, they must have training and ethics. (Woman, aged 66, rural area resident) 

The focus group participants considered accessibility and availability to be crucial qualities in a link worker. Participants wanted a link worker who was easy to reach (e.g. responsive to calls or emails), with whom appointments were quick to schedule (i.e. not a long multi-week or -month wait time), and who were available for follow-up and a continued long-term relationship. They didn’t want to reintroduce their story and meet someone new at every appointment, and would rather have a long-standing relationship with one link worker or a stable team of link workers. 

Most people don’t want to wait a week and a half or six weeks for an appointment with a link worker. If somebody could call them back within the day, and then they could organize a longer appointment or longer stay, and they could, you know, realize you one way or another. But I think availability is, without that you got nothing. You drop the ball if you make people wait too long. (Woman, aged 70, small city/town resident) 

Finally, link workers who prioritized a person-centred approach were essential. Participants wanted a link worker who was willing to try to understand their entire life circumstances, understand how they may have ended up where they are, and what unique needs they may have as a result. They wanted a link worker who equally respected their dignity and autonomy in personal decision-making. They did not want someone to tell them what to do or make assumptions about them without trying to understand first. Inevitably, a link worker who prioritizes a person-centred approach will also make efforts to prioritize individualized care. 

Just the understanding they are there to help us, not save us, not rescue us. I need to have options presented to me, and me to be free to decide where I go from there. And it’s not a failure to me or to them if I don’t like what’s being proposed. (Man, aged 64, large urban centre resident) 

For some individuals, this meant someone with similar life experiences, especially similar age, was preferred. Others, however, were satisfied with a link worker who demonstrated understanding, regardless of age or life experience. As one participant said, “I don’t think it’s entry level. I think it requires some depth” (woman, aged 64, rural area resident).

***5. Barriers to participation***

The primary barriers to participating in SP were transportation, accessibility and financial constraints. Lack of reliable public transportation, difficulties driving, long commutes and limited mobility were cited as barriers to accessing SP services, let alone the initial SP appointment: “I can only go as far as I walk. That’s a huge barrier for me, like even just to make an appointment with the doctor is quite an experience, because I’ve got [to] arrange rides and all that kind of stuff” (woman, aged 66, rural area resident). Many individuals, being retired, found that the cost of many activities of interest could limit their participation, even with subsidization.

Service availability, particularly in rural locations, was a clear external barrier:

Well, there’s not much here. That where I live and in other areas outside of urban areas are kind of neglected when it comes to any kind of programs, because, well, this is no way to deliver them, because there isn’t the population. (Woman, aged 69, small city/town resident) 

Language barriers for those who were non-native English speakers or relied more on nonverbal communication styles (e.g. those with dementia), were also raised.

Older adults worried that there would be a lack of continuity of care in SP programs. They outlined the importance of care providers being reachable, open to follow-ups, and accessible, and of SP being provided in a manner as helpful to them and as reliable as possible:

When a person is asking and reaching out for help, you grab them while they’re willing to give it a chance. On the spot. Don’t wait, because it may have been the only time they’ll actually ask you for help. And in the meantime, you may just end up losing them. (Woman, aged 65, small city/town resident) 

Rigid eligibility criteria for certain programs and services were another barrier to participation. For instance, cut-offs by age may not address the actual need and may limit older adults’ ability to access services. 

Participants with health issues or disabilities experienced difficulties due to all of the aforementioned barriers. However, some individuals with severe disabilities often cited specific barriers, such as the toll that continuously asking for help could take on their dignity, and the need for providers to understand and empathize with the unique day-to-day changes in their ability to take care of even the simplest of activities. 

There will be events here that I would love to go to, but I can’t afford it. And I stopped asking for waivers because it’s just too hard and embarrassing. You’re kind of giving somebody else control over what you want to do. It’s like asking permission and it’s not a good feeling. (Man, aged 58, small city/town resident) 

Further internal barriers, including their current isolation and subsequent sense of social anxiety, were also brought up as huge barriers in preventing them from seeking out SP options, even though they knew SP could help them. For example, feeling embarrassed and “othered” for needing SP services could be a significant barrier. As one participant expressed, “It might be difficult to reach out for help if your problem is embarrassing or not socially accepted” (woman, aged 61, rural area resident).

The stigma experienced by participants and the ageism among providers and link workers in the SP process were identified as barriers as well. Some participants felt that they had been put into a box, or fit into a template, in the minds of providers, causing providers to miss opportunities to connect and truly understand participants’ life experiences, needs and desires: 

My experience with my doctor is, she’s young, and she makes assumptions about what I’m like at my age, and she’s completely wrong. She sees me as an old lady. And I am. But there’s more to me than that. And I think for young people, that’s all they see. They see the white hair, and they make assumptions about who I am and what I might like. I want somebody who would understand my interest, my capabilities, rather than lump me into a group with old people just because we’re all old people. (Woman, aged 81, small city/town resident) 

Finally, the constant need to self-advocate was brought up and outlined two major concerns: (1) those unable to self-advocate risk being overlooked and forgotten; and (2) for those who can, constant self-advocacy can be exhausting: 

The most patient people are most likely to fall between the cracks are the quietest and shyest, and I don’t know what the system of SP is going to do here, but we have to overcome the reluctance people have because they simply don’t feel comfortable talking to strangers or burdening people with their own issues. (Man, aged 78, rural area resident) 

## Discussion

Our study assessed the SP needs and priorities of socially marginalized and structurally disadvantaged Canadian older adults. A total of 10 focus groups were conducted with 3 to 7 participants each, for a total of 43 participants. The general goal of the focus groups was to identify older adults’ current experience with SP, willingness to participate and interest in various SP activities, and comfort bringing up social needs to a primary health care provider or other community providers, and the qualities they would like to see in a link worker, as well as any barriers, real or perceived, they might face that would prevent them from participating in SP. 

Although some participants knew of SP, many were unaware of the term, suggesting room for efforts to increase awareness of SP. Our findings reflect those of similar research indicating the need to promote awareness of SP. For example, although there is little research exploring public awareness of SP in Canada, research from England suggests that there is limited awareness among the public of what social prescribing entails, and indicates the relevance of awareness for uptake of SP; if individuals do not know what SP is, they are highly unlikely to participate.^29^

In asking about participants’ comfort discussing social needs with their health care provider, discussions ultimately turned towards their overall relationship, indicating the importance of client–provider relationships if SP is to work. Even among older adults who felt they had a positive relationship with their health care provider, discussing social needs still felt like an uncomfortable and unfamiliar topic, suggesting the need to normalize conversations. This finding further suggests that the success of having a conversation about loneliness, rather than purely medical topics, is highly contingent on the personal relationship someone has with their health care provider, and the societal awareness of the concept at large. Our findings are aligned with those of a recent systematic review, which found that one of the key implementation factors for SP success among older adults was their relationships with health care providers and link workers. These researchers found that older adults who felt reassured, encouraged and comforted by their health care provider or link worker throughout the process were more likely to have positive experiences and outcomes in SP.^17^

Additionally, varying types of barriers and differences in personal identity were described that may foster or hinder participation, outlining the importance of a person-centred approach to SP. One potential solution to limiting the impact of barriers older adults may face is to continue to prioritize the co-designing backbone of SP.^16^ This may be accomplished by including the opinions of older adults in every stage of the SP framework development in Canada, as well as co-designing each individual prescription to tailor it to the unique needs and priorities of each client.

Underlying these topics, the three themes of SDT—autonomy, relatedness and competency—were important for understanding why older adults had the feelings and opinions that they did. First, it was important that their autonomy was respected, such as through interactions with their primary health care providers in decision-making. Second, there was a strong desire to gain a sense of relatedness (i.e. connectedness and belonging) with their community but also with the providers and link workers who are part of the SP pathway in the form of trust, genuine care and empathy. Third, older adults wanted a sense of competency and influence over which SP programs they would participate in, the outcomes they might have and what SP might offer them. 

Our study contributes to the field of SP by presenting views of Canadian older adults from multiple life circumstances who are structurally disadvantaged and socially marginalized. Future focus groups can explore more deeply the values identified in this study, and how SP can be curated to fit the personalized needs of each individual. 

***Strengths and limitations***

Our study had several strengths. Firstly, the use of qualitative focus groups provided an opportunity for in-depth examination and exploration of participants’ experiences and perspectives, which is often missed in quantitative data. The use of groups allowed for the inclusion of multiple voices, fostering rich discussion. The semistructured nature provided flexibility and adaptability, allowing the direction of the discussion to be influenced by the participants themselves, as the experts of their own experiences. This allowed emerging themes to be explored, such as the need to be proactive, as they were raised by participants. Finally, an online platform made it feasible to reach participants across Canada (including rural and urban participants), as well as those with mobility issues unable to attend an in-person session. This ensured a diverse and geographically representative sample. 

However, our study also had several limitations. First, while efforts were made to include a diverse sample, we relied on a nonrepresentative, opt-in online recruitment method that may have introduced bias into our sample. Second, while we aimed to be inclusive, some participants may have been unable to participate due to our use of an online platform, which not all older adults may be comfortable using.^30^ Third, themes unique to specific demographic groups could not be parsed out, given that each focus group was a mix of participants with multiple forms of experience. 

However, additional focus groups were hosted for minority groups only, in order to identify potential minority-specific attitudes and thoughts toward SP. These additional interviews demonstrated that the underlying themes and comments were consistent across all groups, with some additional considerations brought forth: the need for cultural competency from providers and link workers; the unique barriers to newcomers, such as language to connect and communicate; and knowledge about resources available to newcomers in Canada. 

In future research, focus groups consisting of specific demographic groups (e.g. those with mobility issues, Indigenous people, those with low income, etc.) would further inform the themes, but could not be undertaken in this study given the nature of the study and the available resources. 

## Conclusion

The aim of our study was to explore the perceptions structurally disadvantaged and socially marginalized older adults may hold regarding SP practices that integrate health and social care and well-being, and what they want from such processes. Our older adult focus group participants suggested they would experience a wide range of potential barriers in accessing social services and barriers in engaging with their providers, and indicated their interests in SP, highlighting the importance of a person-centred approach to SP programs. Furthermore, SP programs that prioritize bolstering older adults’ sense of autonomy, relatedness and competency are integral to tailoring SP programs to the needs and priorities of older adults. We suggest that SP developers prioritize client participation in the co-designing and co-production of SP programs to ensure they meet the needs of the unique population whom they serve. 

## Acknowledgements

The Public Health Agency of Canada and Canadian Red Cross provided funding for this study. KGC is supported through a Scholar Award from Michael Smith Health Research BC. 

## Conflicts of interest

The authors declare there are no conflicts of interest.

SA, KM and KGC are Guest Editors of this special issue, and therefore recused themselves from the review process for this article.

## Authors’ contributions and statement

CY: conceptualization, methodology, data curation, formal analysis, project administration, writing—original draft, writing—review and editing.

SL: data curation, project administration, writing—review and editing.

KGC: conceptualization, methodology, funding acquisition, supervision, writing—review and editing.

SB, PH, SH, KM, MN, MS, VW: conceptualization, methodology, writing—review and editing.

SA: writing—review and editing.

The content and views expressed in this article are those of the authors and do not necessarily reflect those of the Government of Canada.
